# Effect of* Melissa officinalis* L. leaf extract on manganese-induced cyto-genotoxicity on* Allium cepa* L.

**DOI:** 10.1038/s41598-023-49699-6

**Published:** 2023-12-13

**Authors:** Ünal Üstündağ, Oksal Macar, Tuğçe Kalefetoğlu Macar, Emine Yalçın, Kültiğin Çavuşoğlu

**Affiliations:** 1 Department of Biology, Faculty of Science and Art, Giresun University, Giresun, Turkey; 2 Department of Food Technology, Şebinkarahisar School of Applied Sciences, Giresun University, 28400 Giresun, Turkey

**Keywords:** Mutation, Plant genetics

## Abstract

Although the antioxidant properties of *Melissa officinalis* extract (Mox) are widely known, little work has focused on its protective capacity against heavy metal stress. The primary objective of this study was to determine the potential of Mox to mitigate manganese (II) chloride (MnCI_2_)-induced cyto-genotoxicity using the Allium and comet assays. Physiological, genotoxic, biochemical and anatomical parameters as well as the phenolic composition of Mox were examined in *Allium cepa* (L.). Application of 1000 µM MnCl_2_ reduced the rooting percentage, root elongation, weight gain, mitotic index and levels of chlorophyll *a* and chlorophyll *b* pigments compared to the control group. However, it increased micronuclei formation, chromosomal abnormality frequencies, tail DNA percentage, proline amount, lipid peroxidation level and meristematic damage severity. The activities of superoxide dismutase and catalase also increased. Chromosomal aberrations induced by MnCl_2_ were fragment, sticky chromosome, vagrant chromosome, unequal distribution of chromatin and bridge. Application of 250 mg/L Mox and 500 mg/L Mox along with MnCl_2_ significantly alleviated adverse effects dose dependently. The antioxidant activity bestowed by the phenolic compounds in Mox assisted the organism to combat MnCl_2_ toxicity. Consequently, Mox exerted remarkable protection against MnCl_2_ toxicity and it needs to be investigated further as a potential therapeutic option.

## Introduction

Manganese (Mn), with an atomic weight of 54.94 g/mol, is the second most common transition metal found near the earth’s crust after iron and is also abundant in biological materials such as soil and sediments^1^. Due to the magnetic properties of pyrolusite ore, it takes its name from the Latin word magnes, meaning magnet^2^. Industrial sources, including coke ovens, power plants, and iron and steel foundries, are the primary Mn emitters to ambient air^3^. Since the middle of the nineteenth century, it has been utilized in the production of steel, non-ferrous alloys, glass, water treatment chemicals, and plant fertilizers ^4^. The foods with the highest amounts of Mn are cereals, vegetables, fruits, nuts, legumes and tea. Drinking water also contains small amounts of Mn at concentrations between 0.001 and 0.1 mg/L^3^. As an essential element for almost all living things, it is necessary for the proper functioning of metabolism as well as for the maintenance of healthy growth and development^3^. Actually, Mn has two crucial roles: it is a crucial cofactor for enzymes and a metal with a catalytic function in biological clusters^5^. Glutamine synthetase, arginase, pyruvate carboxylase and SOD are some of the important enzymes which require Mn as a metal cofactor^6^. Furthermore, the metabolism of carbohydrates, proteins, amino acids and lipids takes place in the presence of Mn. Mn is an important element for many key biological functions, including the synthesis of bone and cartilage, production of vitamin B and vitamin C, functioning of the urea cycle, mitochondrial maintenance, antioxidant defense, hematopoiesis, brain development, endocrine regulation, glucose generation and wound healing^7,8^. In plants, Mn at levels of 20–40 mg per kg of dry weight is vital as it is involved in various metabolic activities such as respiration, photosynthesis, enzyme activation and protein and fatty acid synthesis ^9^. Even though Mn deficiency is uncommon, overexposure to this metal can result in toxicity^10^. Contaminated food, drinking water, air, dust, soil or groundwater can all lead to high Mn exposure^8,11^. In addition to its genotoxic, neurotoxic, cardiotoxic and hepatotoxic effects, Mn is a cytotoxicity inducer and this last effect is caused by apoptosis triggered in Mn-accumulating cells^12,13^. Mitochondria and endoplasmic reticulum malfunctions have an important role in the mechanism of Mn toxicity. High levels of reactive oxygen species (ROS) within the mitochondria and increased calcium influx, which leads to enhanced permeability of the membrane, are the main causes of mitochondrial dysfunction in Mn-exposed cells^7,12^. Excessive exposure to Mn induces oxidative stress, inhibits enzyme activity, limits the absorption and translocation of mineral components, suppresses chlorophyll synthesis and blocks photosynthesis in plants^1^.

*Allium cepa* L. stands out from other plant models due to its huge size and few chromosomes (2n = 16), which makes it easier to assess genotoxicological (DNA damage, micronuclei and chromosomal aberrations) and cytotoxicological (mitotic index) characteristics^14^. The Allium assay examines genomic alterations that result in morphological changes in chromosomes rather than basic point mutations. Observable characteristics, including macromorphological results (slow growth, limited root production and root elongation, loss of chlorophyll) and root anatomical defects, can also be easily included in the test because they are very accessible^15,16^. The test system also produces results that are comparable to those of other eukaryote test systems, minimizes the use of animal species in experimentation and does not require ethical approval, all of which have considerable benefits^17,18^. The range of applications of this test in environmental monitoring has steadily expanded, as it has been used for a large number of chemicals to date. Besides being a cheap and simple system, the Allium assay has advantages over other short-term assays that require exogenous metabolic system addition and prior sample preparation^19^. The comet test, which detects genetic damage at the DNA level, has gained a lot of popularity in recent years as a highly sensitive and reliable test similar to the Allium assay and has also been adapted to the *A. cepa* model^18^. The genotoxicity of compounds in prokaryotes and eukaryotes, as well as the environmental monitoring of these substances, have both been studied for 30 years using this test^20^. Nowadays, the comet assay is acknowledged as a highly advantageous approach because of its ease of use, adaptability, speed and visibility in assessing DNA damage and repair in individual cell populations both quantitatively and qualitatively^21^. Additional benefits of the comet assay include its sensitivity in identifying low amounts of DNA damage, its minimal cell count requirement (less than 10,000) per sample and its ability to utilize both proliferating and non-proliferating cells^22^.

*Melissa officinalis* L., a member of the *Lamiaceae* (mint) family, is a perennial medicinal plant used in traditional medicine around the world. The plant, also known as lemon balm, balm mint and honey balm, is naturally grown in the Mediterranean Region and Western Asia and is now cultivated all over the world^23^. *M. officinalis* is used to treat a variety of conditions in traditional medicine, including sleep disorders, hysteria, nervous agitation, melancholia, chronic bronchial catarrh, migraine, bell palsy, halitosis, toothache, headache, high blood pressure, rheumatism, nerve pain, epilepsy, paralysis, arthritis and mastitis^24^. Extracts from *M. officinalis* leaves are also known to have antioxidant, antiviral, antiparasitic and antibacterial properties^25^. The medicinal and curative qualities of *M. officinalis* are associated with the abundance of volatile molecules, triterpenes, phenolic acids, flavonoids and other beneficial phytochemicals^26^. The phenolic compounds, such as caffeic, gallic, rosmarinic, ferulic and chlorogenic acids, as well as quercetin and rutin, are among the significant bioactive components of *M. officinalis*^27,28^.

As a result of industrialization, health problems due to exposure to heavy metals are becoming serious, and the side effects of drugs used against them push people to seek preventive remedies from nature. Even though *M. officinalis* has been the subject of numerous studies about its potential to be both antigenotoxic and anticytotoxic, there is limited information available in the literature regarding its preventive effectiveness against heavy metal toxicity. This study differs from previous studies by investigating the effectiveness of *M. officinalis* leaf extract (Mox) against MnCI_2_-induced toxicity from a multifaceted perspective in a eukaryotic organism that is highly compatible with human tests. Therefore, the main goal of this work was to show whether Mox could mitigate MnCl_2_-induced toxicity by employing both the comet test and the Allium test in tandem. Changes in antioxidant enzyme activities (SOD and CAT), cell membrane damage, chlorophyll and proline amount and effects on growth were investigated in addition to micronuclei (MN), mitotic index (MI), chromosomal aberrations (CAs) and DNA damage parameters. The phenolic compounds contained in Mox were analyzed in order to interpret the basis for the efficacy of *M. officinalis*.

## Materials and methods

**Figure 1:**
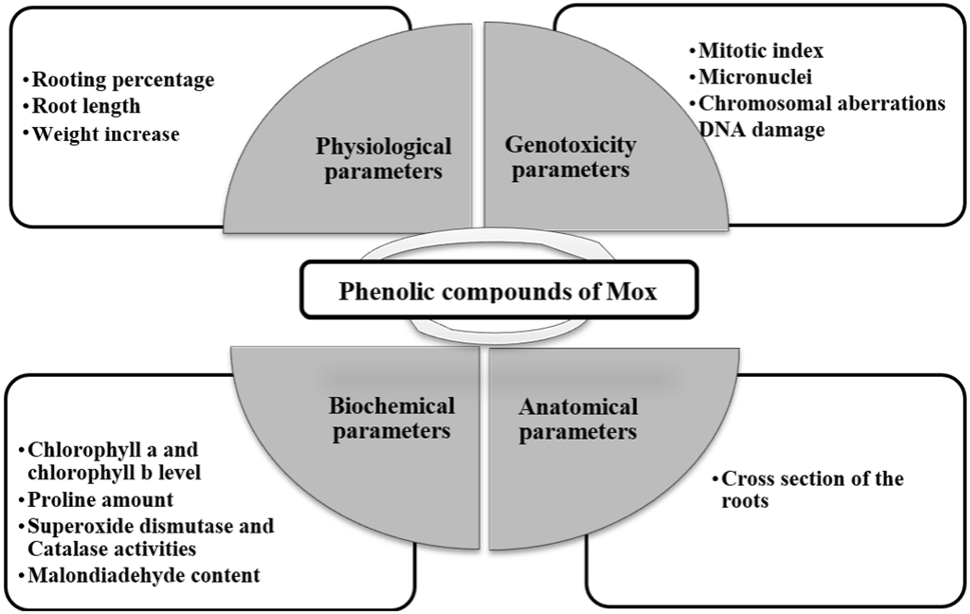
Analyses at the end of the experimental period.

Onion bulbs of almost the same size purchased from Giresun, Türkiye, were dedusted in the laboratory. The disc stem was cleared of any dried roots since it would be in touch with the solutions. Experimental research and field studies on plants and plant parts (Onion bulbs, *M. officinalis*), including the collection of plant material, comply with relevant institutional, national, and international guidelines and legislation. Mn solution was prepared using the chemical manganese (II) chloride (MnCI_2_) [Merck (CAS No: 7773-015)]. The selected MnCI_2_ dose was based on the previous study of Tümer et al.^29^. Mox doses were selected based on preliminary studies considering growth parameters. *M. officinalis* samples were collected in June 2023 in Giresun (40°32′89.2" N; 38°43′48.1" E), Turkey. Identification of *M. officinalis* was made in the Department of Botany of Giresun University (Gaziler District, Prof. Ahmet Taner Kışlalı Avenue, postal code: 28200 Giresun/Türkiye) according to the “Flora of Turkey”^30^. A specimen of the plant was archived in the herbarium with the voucher number BIO-Moff212/2023. Handpicked leaves were air dried in the dark room for 7 days at room temperature. Dried leaves were mechanically powdered using a blender. Two gr of ground leaf samples were extracted with 100 mL of methanol in a mechanical stirrer for 48 h. After filtration (Whatman no. 4), the extract was dried with a rotary evaporator (Heidolph, Hei-VAP ML, Germany) operating under vacuum. The yield of the Mox extract was determined to be 2.1%. Preliminary studies were carried out for the preferred doses of *M. officinalis*. Six groups of Allium bulbs (n = 50) were created, one of which served as the control group and was treated with tap water during the entire experiment. The other groups were subjected to 250 mg/L Mox, 500 mg/L Mox, 1000 µM MnCl_2_, 1000 µM MnCl_2_ + 250 mg/L Mox and 1000 µM MnCl_2_ + 500 mg/L Mox solutions, respectively, in the dark for 3 days. The part of the bulbs in contact with the solutions was the basal plate, where new roots would appear. The solutions in the tubes were freshened every day. Figure 1 summarizes the analyses conducted at the conclusion of the experiment.

### Analysis of phenolic compounds in Mox

Phenolic compounds have an important role in the antioxidant power of plant extracts. The quantitative phenolic component composition of Mox was determined by using high-performance liquid chromatography coupled with tandem mass spectrometry (LC–MS/MS) analysis, which is a sensitive and precise approach for metabolite determination. The analysis was completed at Hitit University, HUBTUAM Laboratory. Before being filtered through a 0.45 m syringe filter for analysis, 1 g of the material was extracted with a 4:1 methanol-dichloromethane solvent in an ultrasonic bath for 2 h. The analysis was carried out using a Thermo Scientific LC–MS/MS instrument (ICAP QC, USA) and an ODS Hypersil column (4.6 × 250 mm). The column oven was set to 30 °C, the solvent flow rate was 0.7 mL/min and the analysis lasted for 34 min. The following are the criteria for the MS/MS analysis: Temperatures of the capillary are 300 °C, the vaporizer is 350 °C, the sheat gas pressure is 30 Arb, the aux gas pressure is 13 Arb, the positive and negative polarities are 2500 V and 4 µA, respectively^31,32^.

### Analysis procedure for physiological parameters

The rooting percentage was calculated for the bulbs whose roots measured 10 mm or longer. The formula used to get the rooting percentage (%) was [(number of rooted bulbs/total number of bulbs) × 100]. A precise balance was used to determine the bulb weights (g) both before and after the experiment. The difference was calculated as a weight increase. Root length (cm) was established with a ruler. Randomly selected bulbs were used in the assessments of root length and weight gain^33^.

### Analysis procedure for genotoxicity parameters

The cytotoxic effects of MnCl_2_ and the mitigating effect of Mox against it were assessed using the MI value, frequency of CAs and MN formations, and the degree of DNA damage (comet assay).

Mitotic slides were prepared from root tip cells at the end of the experiment period in order to determine the MI value, CAs and MN formation frequency. The roots of bulbs exposed to MnCl_2_ and Mox solutions were cut into 1 cm pieces, fixed in Clarke fixative for 2 h and washed thoroughly with distilled water. Root tips were kept in 1 N HCl at 60 °C for 12 min to hydrolyze and then washed again^34^. Then, after the root tips of the samples were stained with acetocarmine for 16 h, the slides were prepared with the standard squash preparation method. This method allowed cells to be examined as a single layer on the slide. The slides were inspected using an IM-450 digital research microscope by IRMECO. For each group, 1,000 cells were counted to determine the MN and CA frequencies. While the formula [MN% = (number of cells with MN) ∕ (total number of cells) × 100] was used to find the MN frequency, the CA frequency was determined by the [CA% = (number of cells with CA) ∕ (total number of cells) × 100] formula. On the other hand, to determine MI for each group, 10,000 cells were counted and the formula [MI = (number of cells undergoing mitosis) / (total number of cells)] was used.

The method recommended by Chakraborty et al.^35^ was followed to conduct comet analysis. Root samples were homogenized in a 400 mM Tris solution in order to isolate the nuclei needed for obtaining nuclear suspension. The slides were covered with a 1% NMPA solution and allowed to air dry for 12 h at room temperature and in the dark. The slide was recoated by combining 40 L of nuclear suspension with 40 L of 1% LMPA. The nuclear suspension-containing slides were put into a horizontal gel electrophoresis tank containing 1 mM Na_2_EDTA and 300 mM NaOH (pH > 13). Electrophoresis was carried out at 4 °C and 0.7 V/cm (20 V and 300 mA) for a period of 20 min after waiting 15 min. The slides were stained with ethidium bromide for 5 min and then photographed using a fluorescence microscope after being neutralized with tris-buffer. The "TriTek 2.0.0.38 Automatic Comet Testing Software" was used to measure the comet sizes. The DNA ratios (%) of head and tail sections were determined. Using the percentage of tail DNA as a reference point, DNA damage was classified according to the comet scale as follows: ≤ 5% indicates none or minor damage, 5–20% indicates low damage, 20–40% indicates moderate damage, 40–75% indicates high damage, and %75 indicates severe damage^36^.

### Analysis procedure for biochemical parameters

All experiments were set up in triplicate to allow for statistical evaluation. Chlorophyll *a* and *b* measurements were performed on bulbs with green leaves. In order to extract pigments, 0.1 g of leaf sample was crushed with a plastic rod in 2.5 mL of an 80% acetone-containing tube in the dark for 7 days^37^. After the mixture had been filtered, 2.5 ml of 80% acetone were added to the tube. Following a centrifugation process at 3000 rpm, the absorbance of the supernatant was measured spectrophotometrically at 645 and 663 nm. The formulae (Eqs. 1 and 2) proposed by Witham et al.^38^ were utilized to compute chlorophyll contents:

1 $${\text{Chlorophyll}} \, a = \, \left( {{12}.{7 } \times {\text{ A663 }}{-}{ 2}.{69 } \times {\text{ A645}}} \right) \, \times \, \left( {{\text{V}}/{1}000 \, \times {\text{ W}}} \right)$$

2 $${\text{Chlorophyll}} \,b = \, \left( {{22}.{9 } \times {\text{ A645 }}{-}{ 4}.{68 } \times {\text{ A663}}} \right) \, \times \, \left( {{\text{V}}/{1}000 \, \times {\text{ W}}} \right)$$

A663 and A645 are the absorbances of the supernatant at 663 and 645 nm; V is the final volume (mL) of 80% acetone with the supernatant and W is the weight of the fresh leaf (g).

To calculate the proline concentration, 0.25 g of root segment was homogenized in 5 mL of 3% aqueous sulfosalicylic acid^39^. After filtering by Whatman No. 2 filter paper, the filtrate was combined with acid-ninhydrin and glacial acetic acid in equal volumes. The tubes containing the mixture were kept in an ice-filled container for two minutes in order to halt the reaction. The mixture was then stirred for 10 s with 2 mL of toluene. The absorbance of the chromophore was measured spectrophotometrically at 520 nm. The proline concentration in fresh samples was determined using a standard graph created from the absorbance of different concentrations of proline solutions (Eq. 3).

3 $$\left[ {\left( {\upmu {\text{g proline mL}}^{{ - {1}}} \times {\text{ mL toluene}}} \right) \, /{ 115}.{5 }\upmu {\text{g }}\mu {\text{mole}}^{{ - {1}}} } \right] \, / \, \left[ {\left( {\text{g sample}} \right)/{5}} \right] \, = \, \upmu {\text{moles proline g}}^{{ - {1}}} {\text{FW}}$$

MDA was examined at the root tip to gauge the extent of cell membrane damage^40^. Freshly cut root tissues weighing 0.25 g were homogenized in a 0.5 mL trichloroacetic acid (TCA) (5%) solution. The homogenate underwent a 10-min centrifugation at 12,000*g*. Equal volumes of the supernatant and thiobarbituric acid (0.5%) were mixed and allowed to react in TCA (20%) at 98 °C for 25 min. The mixture-containing tubes were put in an ice-filled container to interrupt the reaction inside the tubes. The mixture underwent a 5-min centrifugation at 10,000*g*. The absorbance of the supernatant was measured spectrophotometrically at 532 nm to calculate the MDA concentration as µM/g FW.

Freshly cut root tissues weighing 0.25 g were homogenized in 2.5 mL of monosodium phosphate buffer (50 mM/pH 7.8) and centrifuged at 10,500*g* for 20 min after being rinsed with distilled water. Following a 20-min centrifugation at 10,500*g*, the enzyme-containing supernatant was used to evaluate SOD and CAT activities^41^. The enzyme extract (0.01 mL) was transferred to a reaction medium (3 mL volume), which was made up of monosodium phosphate buffer, nitroblue tetrazolium chloride, methionine, riboflavin, EDTA-Na_2_, insoluble polyvinylpyrrolidone and distilled water to detect SOD activity. The tubes containing the mixture were exposed to two 15 W fluorescent lamps for 10 min for the enzymatic reaction to take place. Following a 10-min period in darkness to terminate the reaction, the absorbance at 560 nm was measured spectrophotometrically^42^. The enzyme extract (0.2 mL) was transferred to a reaction medium (2.8 mL volume), which was made up of hydrogen peroxide (H_2_O_2_), monosodium phosphate buffer and distilled water to detect CAT activity. The decrease in absorbance at 240 nm indicating enzymatic elimination of H_2_O_2_ was monitored spectrophotometrically^43^. The units of enzyme activities were calculated as U/mg FW for SOD and OD_240_ nm min/g FW for CAT.

### Analysis procedure for root meristem anatomical defects

In order to understand the effects of MnCl_2_ application on root meristem anatomy, cross-sections taken from onion roots from each group were investigated. Sections were taken manually with a razor blade, stained with methylene blue (1%) and photographed immediately using an IRMECO, IM-450 TI research microscope.

### Statistical analysis

Both Kolmogorov–Smirnov and Shapiro–Wilk anomaly tests were applied to all data belonging to the six different groups created, and as a result, it was determined that all data had a normal distribution (*p* > 0.05). The statistical analysis tool "IBM SPSS Statistics 23" was used to statistically examine the results of the study. Results are presented as mean ± SD (standard deviation). One-way ANOVA and Post hoc Multiple Comparisons Duncan tests were used to examine the statistical significance between the data of each group. The threshold for statistical significance was selected at *p* < 0.05.

## Results and discussion

**Figure 2:**
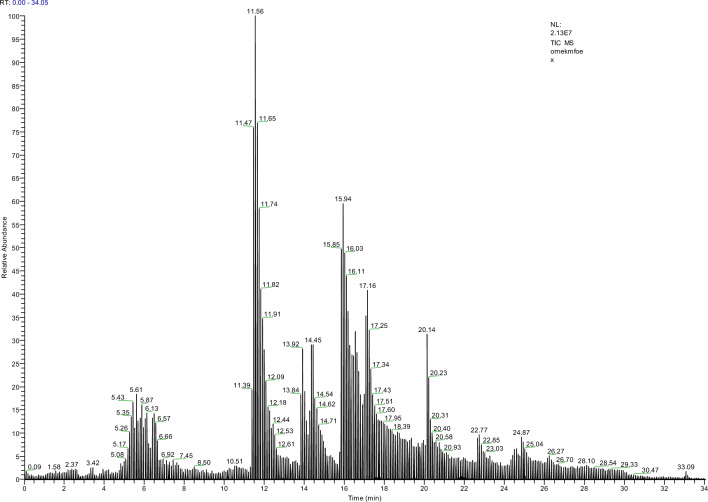
LC–MS/MS chromatogram of Mox.

**Figure 3:**
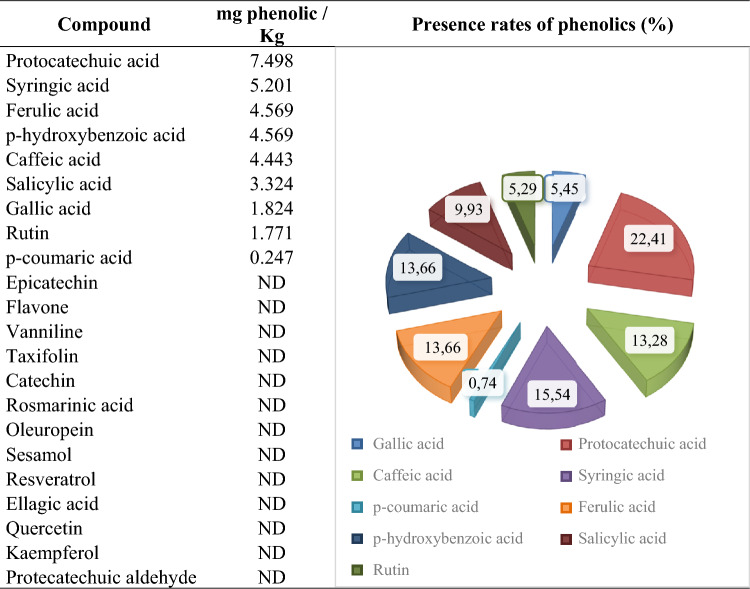
The abundance of phenolic compounds in Mox. ND: not detected.

The fact that consumption of fruits and vegetables reduces the risk of chronic diseases and that natural antioxidants taken from the diet are safer than their synthetic counterparts increase interest in natural plant extracts^44^. The bioactive compounds found in herbal extracts are directly related to their protective effects against chemicals. The phenolic content of *M. officinalis* was identified using LC–MS analysis in order to understand the protective potential of Mox against MnCl_2_ (Figs. 2, 3). *M. officinalis* contains several antioxidants, especially those from the phenolic compound family. The most prevalent phenolic compounds in Mox were protocatechuic acid (22.41%), syringic acid (15.54%), *p*-hydroxybenzoic acid (13.66%), ferulic acid (13.66%), caffeic acid (13.28%), salicylic acid (9.93%), gallic acid (5.45%), rutin (5.29%), and p-coumaric acid (0.74%). Oniga et al.^45^ identified that the aerial parts of *M. officinalis* contain ferulic acid, caffeic acid and p-coumaric acid. Furthermore, a HPLC investigation by Arceusz and Wesolowski^46^ also revealed that *M. officinalis* includes syringic, ferulic, chlorogenic, caffeic, gallic and rosmarinic acids. High levels of ferulic, *p*-coumaric, caffeic, chlorogenic, ursolic, rosmarinic and oleanolic acids, as well as tannins and flavonoids, all of which have antioxidant properties, play a significant role in the activity of Mox against the aging process and degenerative diseases due to oxidative stress^47^. Basar and Zaman^24^ reported that *M. officinalis* leaves contain protocatechuic acid, which is the most dominant phenolic in our study. Protocatechuic acid offers a variety of pharmacological functions, such as antioxidant, anti-inflammatory, antibacterial, antiviral, neuroprotective, anticancer, analgesic, anti-osteoporotic and antiaging properties^48^. Syringic acid, the second most prevalent phenolic component in Mox, was shown to have the ability to scavenge free radicals by using DPPH analysis^49^. According to Karamac et al.^50^, due to the two methoxy moieties connected to the aromatic ring, syringic acid is more effective at scavenging free radicals than *p*-hydroxybenzoic acid, the third most abundant phenolic compound in our study. The regulation of *p*-hydroxybenzoic acid under stress conditions is crucial for the antioxidative system since it involves the biosynthesis of salicylic acid and also increases the impermeability of the cell wall, leading to increased resistance^51^. Srinivasulu et al.^52^ suggested that SOD and CAT are among the molecular targets of syringic acid in case of oxidative imbalance. Syringic acid suppresses oxidative stress by modifying the activity of these antioxidant enzymes. de Abreu et al.^53^ reported that caffeic and syringic acids, presumably because of their antioxidant characteristics, reduced the genotoxic impact generated by snake venom. Our findings indicate that ferulic acid is another abundant component of Mox. Ferulic acid can remove surplus ROS or deactivate free radical-producing enzymes directly to prevent oxidative damage^54^. According to Balakrishnan et al.^55^, ferulic acid's capacity to scavenge ROS significantly reduces the genotoxicity that 7,12-dimethylbenz(a)anthracene causes in golden Syrian hamsters. Our research supported the findings of Caniova and Brandsteterova^56^, who demonstrated the rich caffeic acid content of Mox using an HPLC chromatogram. Mox has been shown to be able to inhibit the peroxidation of polyunsaturated fatty acids in relation to phenolic acids such as protocatechuic acid and caffeic acid^57^. Salicylic acid, one of the endogenous plant growth regulators that we have also demonstrated to be abundant in Mox, plays a significant role in plant germination, growth and development by influencing metabolism, photosynthesis, stress control and enzyme activities^58^. It has been established that gallic acid, a different phenolic component of the Mox in our study, acts as a genetic defender by lowering the frequency of MN and preventing DNA chain breaks^59^. Due to the distinctive organization of all these compounds, it is reasonable to remark that Mox is a highly valuable antigenotoxic and anticytotoxic material from nature.

Table 1 presents the impacts of MnCl_2_, Mox and MnCl_2_ + Mox treatments on growth of *A. cepa* bulbs. The C, Mox 1 and Mox 2 groups demonstrated a 100% rooting rate. In light of this, it could be interpreted that the Mox dosages we selected have no influence on root emergence. This suggestion was reinforced by the lack of a significant difference between the root length or weight gain data of the control group and the Mox 1 and Mox 2 groups. However, rooting dropped (69%) in the MnCl_2_-treated group. Similarly, the average root length and the average weight increase of the MnCl_2_-treated group were significantly lower (*p* < 0.05) than those of the control group, respectively. Elevated concentrations of metals and other chemicals impair germination, development, and production processes primarily by affecting the physiological, biochemical, and genetic components of the plants^60^. Millaleo et al.^61^ reported that Mn is both an essential and toxic metal for plants, depending on the dose of exposure. According to Li et al. ^1^, the reduction of plant growth is the most obvious toxicity symptom of Mn. It has been previously demonstrated that Mn suppresses germination and growth in *A. cepa*^29^. Our results are also in agreement with the work of Abou et al.^62^, who evaluated that the Mn-accumulating plant *Alyssum murale* experienced shorter roots and shoots as well as a decrease in chlorophyll concentration in response to higher Mn concentrations. In addition, it was demonstrated that excessive Mn administration induced the development of shorter roots in ryegrass and white clover^63^. Although excess Mn restricts growth in seedlings, especially by inhibiting photosynthetic pigment biosynthesis and photosynthesis, inhibition of the uptake of elements and reduction of auxin accumulation in newly developing roots may be effective in slowing growth^64^. The values of the growth parameters increased notably (*p* < 0.05) depending on the amount of Mox added to the mixture of MnCl_2_ and Mox. The majority of studies have concentrated on the capacity of Mox to inhibit the growth and proliferation of cancer cells. Mox has been demonstrated to have specific toxicity in tumor cells while causing no harm to healthy cells^47^. This is the first study to demonstrate that Mox can significantly revive growth in MnCl_2_-stressed plants. However, it was shown that the growth repressed under heavy metal stress in *Brassica juncea* increased dose-dependently with increasing salicylic acid concentrations, which were found to be plentiful in Mox^65^. Mox has been proven to reduce the intracellular generation of radical molecules^66^. Functional mechanisms of Mox in growth regeneration may include its ability to suppress ROS production, maintain healthy membranes and guard against genetic perturbations that can cause cell death.

**Table 1:** Impacts of MnCl_2_, Mox and MnCl_2_ + Mox on physiological parameters.

Groups	Rooting percentage (%)	Root length (cm)	Weight increase (g)
C	100	7.00 ± 0.95^a^	+ 5.25^a^
Mox 1	100	7.10 ± 0.92^a^	+ 5.07^a^
Mox 2	100	7.40 ± 0.97^a^	+ 4.96^a^
MnCl_2_	69	2.80 ± 0.54^d^	+ 1.54^d^
MnCl_2_ + Mox 1	75	3.70 ± 0.61^c^	+ 2.30^c^
MnCl_2_ + Mox 2	83	4.90 ± 0.74^b^	+ 3.45^b^

C: Control, Mox 1: 250 mg/L *M. officinalis* leaf extract, Mox 2: 500 mg/L *M. officinalis* leaf extract, MnCl_2_: 1000 µM MnCl_2_, MnCl_2_ + Mox 1: 1000 µM MnCl_2_ + 250 mg/L *M. officinalis* leaf extract, MnCl_2_ + Mox 2: 1000 µM MnCl_2_ + 500 mg/L *M. officinalis* leaf extract. Data are displayed as mean ± standard deviation. n = 50 for rooting percentage and, n = 10 for root length and weight increase. Different letters (a–d) in the same column indicate significant differences (*p* < 0.05) between groups.

**Figure 4:**
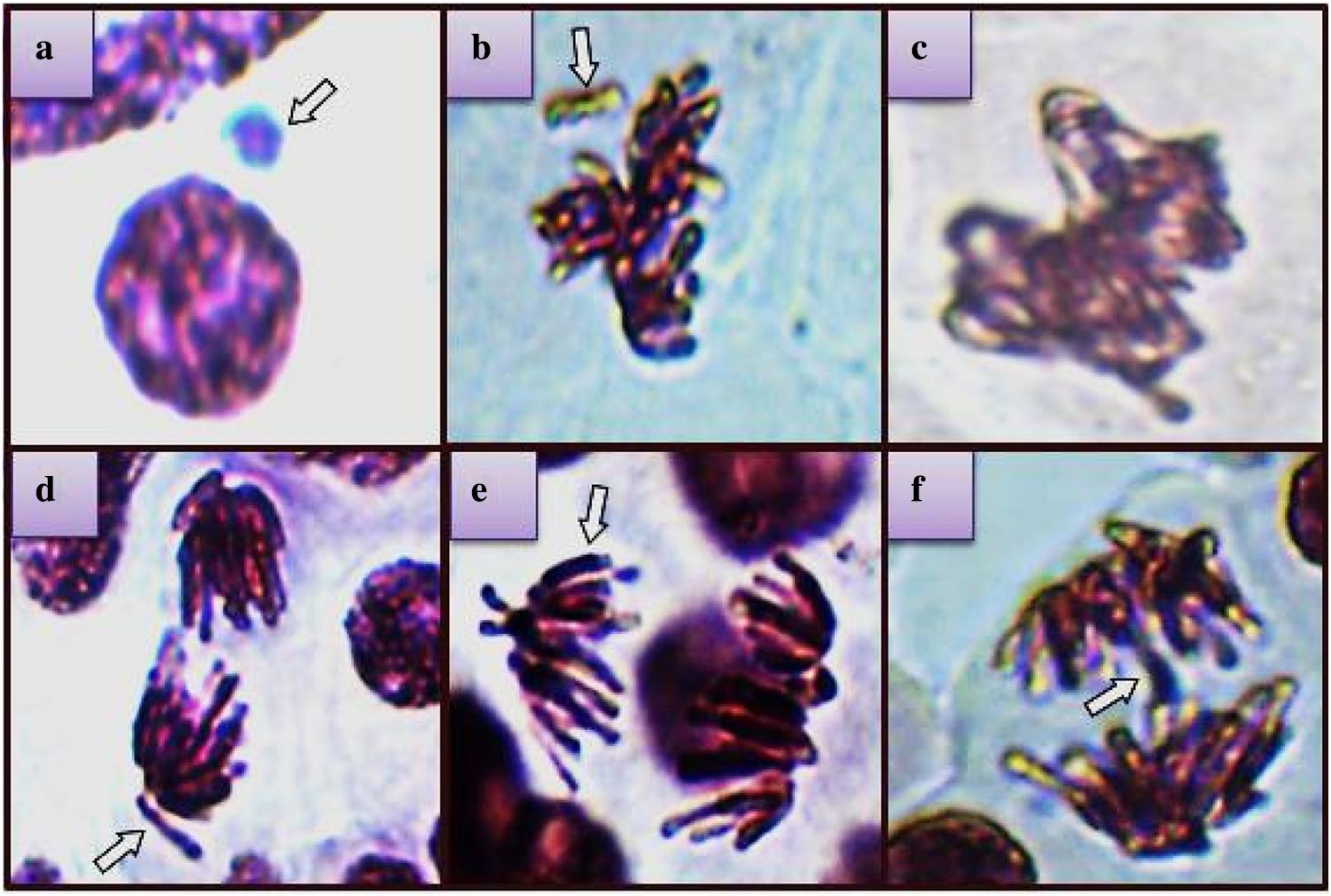
MnCl_2_-induced CA types indicated with arrows. MN (**a**), fragment (**b**), sticky chromosomes (**c**), vagrant chromosomes (**d**), unequal distribution of chromatin (**e**), bridge (**f**).

Figure 4 depicts the CA types in *A. cepa* root cells exposed to MnCl_2_, while Table 2 provides the quantification of genotoxic events. The amounts of MN, MI, and CAs did not differ statistically in the C, Mox, and Mox 2 groups. Therefore, the Mox doses used in the study were not genotoxic for *A. cepa* root cells. On the contrary, the MI of the MnCl_2_ group fell by 24% as a result of the MnCl_2_ treatment. CA types formed in the MnCl_2_ group were listed as MN (63.8 ± 5.56), fragment (45.6 ± 3.95), sticky chromosomes (35.7 ± 3.26), vagrant chromosomes (33.1 ± 2.98), unequal distribution of chromatin (20.9 ± 1.83) and bridge (12.5 ± 1.10) according to their abundance (Table 2). MI is a parameter that measures sample toxicity by monitoring cell division. The findings of our study were consistent with those of earlier studies that found Mn exposure reduced MI in *A. cepa* meristematic cells^29,67^. The fact that MnCl_2_ treatment led to a growth retardation in addition to an MI decrease suggests that Mn inhibits cell proliferation. According to Doroftei et al.^68^, *A. cepa* reacts to MnCl_2_ directly by inhibiting root growth as a result of diminished mitotic division at the root apex. The development of micronuclei (MN) is widely employed as a cytogenetic marker of chromosomal degeneration, genomic instability and ultimately cancer risk^69^. According to Kwon et al.^70^, chromosome fragments or chromosomes that do not fit into the nucleus of the daughter cell during telophase may be the primary source of MN formation. Previous studies have demonstrated that Mn exposure causes MN development, supporting our findings^71,72^. Mn has the potential to be hazardous, as shown by the fact that it can result in chromosomal breakage or modifications to the mitotic spindle during the cell division process. A MN may develop as a result of biological, physical or chemical factors interacting with the centromere and mitotic spindle, changing the mitotic apparatus and impairing chromosomal segregation. Mn perturbs DNA and disturbs the harmony of genetic replication^73^. Fragment was one of the most common CAs in *A. cepa* root cells treated with MnCl_2_ (Fig. 4b). Double-strand DNA breaks or suppression of DNA synthesis lead to the formation of fragments and fragmentation is usually accompanied by MN formation^74^. Another frequent CA type was sticky chromosome in the MnCl_2_ group (Fig. 4c). Stickiness results from either enhanced chromosomal contraction or condensation or from DNA depolymerization and partial breakdown of nucleoproteins^75^. It is a specific type of chromosomal abnormality that prevents mitotic division by binding together several chromosomes^76^. Hussein^77^ pointed out MN induction and stickiness in *A. cepa* as the most prominent indicators of cytotoxicity. The toxic consequences of sticky chromosomes are typically permanent and may cause cellular death^75^. In this case, it can be said that Mn not only suppresses cell division but also has the potential to kill cells. The high proportions of vagrant chromosomes (Fig. 4d) and unequal distribution of chromatin (Fig. 4e) indicate that Mn may be a spindle poison. Yildiz and Arikan^78^ suggest that formation of vagrant chromosomes gives rise to daughter nuclei with unequally distributed chromosomes. According to Tinna^79^, any damage to the pole and the pole determinants would produce an unequal distribution of chromatin. Bridge (Fig. 4f) is an aberration that occurs during the anaphase stage of mitosis and is defined by the existence of chromosomal links across the division planes^76^. Our findings on CAs were consistent with the previous study of Tümer et al.^29^, who demonstrated the accumulation of the same types of CAs by Mn in the same model organism. Furthermore, our results on genotoxicity aligned with those of Seth et al. ^80^, who demonstrated that a different heavy metal, cadmium, was connected to cytogenetic end-points such MI, MN, CAs, and aberrant mitosis. In addition to producing oxidative stress, Mn may build up inside of cells, having cytotoxic impacts and damaging cells. The principal intracellular degenerations brought on by Mn-induced alterations in enzyme and gene expression include DNA helix breakage, chromosomal damage and lipid peroxidation^68^.

**Table 2:** Protective role of Mox against MnCl_2_ genotoxicity.

Aberrations	C	Mox 1	Mox 2	MnCl_2_	MnCl_2_ + Mox 1	MnCl_2_ + Mox 2
MI	810 ± 25.6^a^	802 ± 24.7^a^	826 ± 26.4^a^	615 ± 17.9^d^	667 ± 18.6^c^	734 ± 20.5^b^
MN (%)	0.12 ± 0.16^d^	0.15 ± 0.20^d^	0.08 ± 0.13^d^	63.8 ± 5.56^a^	48.7 ± 4.16^b^	30.8 ± 2.96^c^
FR (%)	0.00 ± 0.00^d^	0.00 ± 0.00^d^	0.00 ± 0.00^d^	45.6 ± 3.95^a^	35.3 ± 3.24^b^	22.4 ± 2.15^c^
SC (%)	0.15 ± 0.21^d^	0.10 ± 0.15^d^	0.00 ± 0.00^d^	35.7 ± 3.26^a^	26.8 ± 2.65^b^	18.9 ± 1.78^c^
VC (%)	0.00 ± 0.00^d^	0.00 ± 0.00^d^	0.00 ± 0.00^d^	33.1 ± 2.98^a^	25.5 ± 2.52^b^	15.6 ± 1.44^c^
UDC (%)	0.14 ± 0.18^d^	0.16 ± 0.21^d^	0.11 ± 0.17^d^	20.9 ± 1.83^a^	14.6 ± 1.25^b^	6.20 ± 0.76^c^
B (%)	0.00 ± 0.00^d^	0.00 ± 0.00^d^	0.00 ± 0.00^d^	12.5 ± 1.10^a^	7.90 ± 0.85^b^	3.10 ± 0.44^c^

C: Control, Mox 1: 250 mg/L *M. officinalis* leaf extract, Mox 2: 500 mg/L *M. officinalis* leaf extract, MnCl_2_: 1000 µM MnCl_2_, MnCl_2_ + Mox 1: 1000 µM MnCl_2_ + 250 mg/L *M. officinalis* leaf extract, MnCl_2_ + Mox 2: 1000 µM MnCl_2_ + 500 mg/L *M. officinalis* leaf extract. Data (n = 10) are displayed as mean ± standard deviation. Different letters (a–d) in the same line indicate significant differences (*p* < 0.05) between groups. 1000 cells (from 10 slides) were counted to determine the MN and CA frequencies. 10,000 cells (from 10 slides) were counted to calculate MI for each group.

*MI* mitotic index, *MN* micronucleus, *FR* fragment, *SC* sticky chromosome, *VC* vagrant chromosome, *UDC* unequal distribution of chromatin, *B* bridge.

The comet analysis was used to assess genomic DNA damage in cells. This test system can accurately identify DNA damages, including single-strand breaks, when cells are exposed to powerful mutagens^81^. According to our findings, there was agreement between the results of CAs and comet tests (Tables 2 and 3). Even though the DNA tail percentage results of C (0.1%), Mox (1.7%), and Mox 2 (0.1%) groups differed statistically, these damages were still categorized as none to minor (≤ 5%). On the contrary, the comet image of the MnCl_2_ group showed that Mn induced notable DNA damage in *A. cepa* root cells (Table 3). Indeed, a DNA tail percentage of 43.1 showed that the MnCl_2_ group had high levels of DNA damage (40–75%). DNA disruption may be attributed to the accumulation of ROS that causes DNA strand breakages and permanent impairments in replication, recombination, repair and transcription^81^. A dose-dependent rise in MI incidence, a decrease in MN and CA frequencies, and a decrease in DNA tail percentages were observed in the MnCl_2_ + Mox 1 and MnCl_2_ + Mox 2 groups. All differences were statistically significant when compared with the MnCl_2_ group. Although the restoration in the genotoxicity findings in these two groups did not reach the levels of the C group, according to the results of the DNA tail percentage, DNA damage fell to a moderate level in the MnCl_2_ + Mox 1 group and to a low level in the MnCl_2_ + Mox 2 group. Similar to our findings, de Carvalho et al.^82^ reported that Mox exerted antimutagenic and antigenotoxic properties in mice, another model organism. Additionally, Oalđe Pavlović et al.^83^ showed that ethanolic, methanolic, and aqueous extracts of *M. officinalis* had anticancer, antigenotoxic, and genoprotective capabilities in various experimental systems. MN, CAs and comet tests also revealed the antimutagenic and anticlastogenic potential of Mox, depending on its therapeutic constituents^84^. Mox owes these virtues to antimutagenic substances such as gallic acid that activate DNA repair enzymes^26^. Indeed, one of the most promising characteristics of genotoxic chemicals is the alteration of DNA repair pathways^85^. Furthermore, protocatechuic acid, the most prevalent phenolic in Mox, has a well-established ability to inhibit oxidative DNA damage and lipid peroxidation. It has been demonstrated that protocatechuic acid affects not only the functions of the enzymes responsible for the metabolism of carcinogens, but also neutralizes reactive intermediate metabolites and so prevents their attachment to DNA^86^. It has also been suggested that *M. officinalis* has the antioxidant potential to keep reactive radicals from harming biomolecules like proteins, DNA, amino acids and polyunsaturated fatty acids due to its ability to donate hydrogen and/or electrons^87^. Lipid peroxidation brought on by oxidative stress is one of the factors that contribute to ongoing DNA and chromosomal damage^88^, and Mox may also be successful at protecting genetic structure by protecting membranes.

**Table 3:** DNA damage induced by MnCl_2_ as determined by comet test.

Parameters	C	Mox 1	Mox 2	MnCl_2_	MnCl_2_ + Mox 1	MnCl_2_ + Mox 2
Head diameter (px)	44.000	40.000	38.000	52.000	62.000	54.000
Head density	322.132	336.002	278.375	386.737	684.136	529.326
**Head DNA (%)**	**99.9 ± 0.32**^**a**^	**98.3 ± 1.49**^**b**^	**99.9 ± 0.32**^**a**^	**56.9 ± 1.20**^**e**^	**78.6 ± 1.90**^**d**^	**90.2 ± 1.62**^**c**^
Tail length (px)	1.000	4.000	2.000	47.000	30.000	14.000
Tail density	306	5.970	123	293.535	185.759	57.657
**Tail DNA (%)**	**0.10 ± 0.32**^**e**^	**1.70 ± 1.49**^**d**^	**0.10 ± 0.32**^**e**^	**43.1 ± 1.20**^**a**^	**21.4 ± 1.90**^**b**^	**9.80 ± 1.62**^**c**^
Tail Moment	0.006752	0.257389	0.001720	20.280	6.406	1.375

C: Control, Mox 1: 250 mg/L *M. officinalis* leaf extract, Mox 2: 500 mg/L *M. officinalis* leaf extract, MnCl_2_: 1000 µM MnCl_2_, MnCl_2_ + Mox 1: 1000 µM MnCl_2_ + 250 mg/L *M. officinalis* leaf extract, MnCl_2_ + Mox 2: 1000 µM MnCl_2_ + 500 mg/L *M. officinalis* leaf extract. Data (n = 10) are displayed as mean ± standard deviation. Different letters (a–d) in the same line indicate significant differences (*p* < 0.05) between groups 1000 cells were counted to determine the DNA damage. The comet scale indicating DNA damage is based on the percentage of tail DNA. None or minor (≤ 5%), low damage (5–20%), moderate damage (20–40%), high damage (40–75%), severe damage (≥ 75%).

Significant values are in bold.

Table 4 displays the impact of MnCl_2_ and Mox treatments on biochemical parameters. Results for chlorophyll, proline MDA, SOD activity, and CAT activity in the C, Mox 1 and Mox 2 groups did not vary statistically. In comparison to the control, MnCl_2_ application statistically altered the results of these parameters. Chlorophyll *a* and chlorophyll *b* concentrations in the MnCl_2_ group decreased by 60% and 76%, respectively, compared to the C group. Our findings are in line with several studies that demonstrate Mn toxicity lowers chlorophyll levels in plants^89,90^. Furthermore, newly emerging leaves of sugarcane exhibited lower chlorophyll levels and chlorosis due to the exceeding Mn exposure^91^. Normal quantities of Mn have a role in both the production of chlorophyll and its defense against photooxidation. On the other hand, excessive amounts of Mn can be readily transported from the roots to the shoot^92^, where it can affect the amounts of photosynthetic pigments. Some enzymes of the isoprenoid biosynthesis pathway, which generates plant pigments, are susceptible to Mn toxicity as well as to Mn deficiency^93^. Additionally, it has been proposed that chlorophyll breakdown brought on by chlorophyll photobleaching or chloroplast photooxidative disturbance is a mediator of excessive Mn-induced chlorosis^94^. According to Arya and Roy^95^, high levels of Mn can harm chlorophyll by causing an iron deficit or by replacing the magnesium needed for the formation of chlorophyll. Indeed, Mn contributes to the development of chloroplasts, thylakoid synthesis and chlorophyll accumulation in leaves^96^. The proline level was 2.69 times higher in the Mn-exposed group than in the control group (Table 4). Plants that are subjected to diverse biotic or abiotic stressors often exhibit an increased accumulation of organic compounds including proline^97^. Our results corroborated the findings of Ragab and Saad-Allah^96^, which showed that Mn administration greatly enhanced proline while drastically lowering growth and photosynthetic pigments. Proline is a crucial component in the defense against Mn stress, as also demonstrated by Pan et al.^98^. Proline has been reported to improve tolerance to Mn by scavenging free radicals and chelating Mn^95^. It is classified as a multipurpose antioxidant that also serves as a cryoprotectant, an osmotic regulator, a metal chelator, and a hydroxyl-scavenger^95^. In contrast to the studies^96^ suggesting that Mn stress raises proline level and decreases MDA amount, both proline and MDA levels rose in the MnCl_2_-treated group in *A. cepa* (Table 4). The MDA level in the MnCl_2_-treated group was 2.91 times that of the control group. Our results concur with the studies showing elevated MDA accumulation in high Mn concentrations in *Lepidium sativum*^99^, *Nicotiana benthamiana*^100^ and *Broussonetia papyrifera*^101^. Giannakoula et al.^102^ suggested that heavy metals could disrupt membrane integrity as well as standard growth in citrus plants. MDA generation serves as both a signal of membrane lipid peroxidation and as proof that ROS damage membranes^101^. According to Morales and Munné-Bosch^103^, the rise in MDA under stressful circumstances can be attributed to the activation of lipoxygenases or polyunsaturated fatty acid peroxidation caused by ROS. Although Mn is known to induce ROS accumulation, plants have the ability to defenestrate high doses of ROS by using a variety of enzymatic and non-enzymatic antioxidants^104^. SOD and CAT are two components of the enzymatic antioxidant system that eliminate superoxide and hydrogen peroxide radicals, respectively. The increase in MDA in the MnCl_2_ group was accompanied by an increase in the activities of SOD and CAT enzymes. The change in MDA level as well as an alteration in SOD and CAT enzyme activities is a definite sign of oxidative stress in plants. The intermediates superoxide radical, hydroxyl and hydrogen peroxide, which are produced as a result of the subsequent reduction of molecular oxygen to water, are potentially hazardous due to their higher reactivity than oxygen molecules^102^. SOD and CAT activities were 3.47 and 2.10 times higher in the MnCl_2_ group compared to the control group, respectively (Table 4). In the literature, there are several studies showing that Mn toxicity multiplied the activities of SOD and CAT enzymes in various plants, including rice^105^, soybean^92^ and onion^29^. Additionally, Zhao et al.^106^ suggested that the SOD and CAT enzyme activities were associated with the Mn tolerance of a Mn-hyperaccumulator plant (*Phytolacca americana*). Our results demonstrated that these enzymes are crucial for the defense of *A. cepa* against Mn toxicity-related oxidative stress. In support of our study, Zhao et al.^106^ reported that an overabundance of Mn causes the accumulation of superoxide and hydrogen peroxide, which are substrates of SOD and CAT enzymes. Mox applied in mixture with MnCl_2_ remarkably increased chlorophyll *a* and *b* levels and remarkably decreased proline and MDA levels in the MnCl_2_ + Mox 1 and MnCl_2_ + Mox 2 groups compared to the MnCl_2_ group (Table 4). Phenolics such as salicylic acid in Mox have been shown to increase the biosynthesis of photosynthetic pigments, including carotenoids that protect chlorophyll in plants under heavy metal stress^65^. Additionally, Xuan and Khang^107^ proved that protocatechuic acid, which was the most prominent phenolic compound in our extract, promoted chlorophyll b synthesis and SOD activity, when applied exogenously. In the MnCl_2_ + Mox 1 and MnCl_2_ + Mox 2 groups, the activities of SOD and CAT enzymes were also significantly lower than those of the MnCl_2_ group. However, these alterations could never reach to the levels of the control group. The protective effect of Mox in the MnCl_2_ + Mox mixtures against Mn toxicity was dose dependent. Therefore, the MnCl_2_ + Mox 2 group outperformed the MnCl_2_ + Mox 1 group in every biochemical parameter. In the literature, studies showing the protective potential of Mox against heavy metal stress are extremely limited. Bilen et al.^108^ reported that SOD activity increased but CAT activity remained constant in *Oncorhynchus mykiss* administered Mox with diet. In another study, Mox supplementation induced an elevation in the SOD and CAT activities in rats^109^. In addition, Martins et al.^110^ found that Mox, because of its antioxidant capabilities, reduced Mn-induced oxidative stress in mice when applied along with Mn and had lower SOD and CAT activity in comparison to the Mn group. Mox is equipped with marvelous bioactive compounds that endow it with extraordinary antioxidant properties. So, both manufactured and organic free radicals can be scavenged by Mox^23^. The antioxidant power of Mox was confirmed through different methods, including DPPH radical scavenging, FRAP, DMPD radical and CUPRAC assays^111,112^. In our investigation, Mox may be able to suppress ROS-mediated oxidative stress under Mn toxicity in order to avoid oxidative damage in vivo. It is important to note that, in comparison to the MnCl_2_ group, the groups treated with the MnCl_2_ + Mox mixture exhibited a drop in genotoxicity along with a decrease in proline levels and enzyme activity and MDA level, indicating a partial cease of oxidative stress. Our study confirms studies showing that genotoxicity induced by abiotic stresses can be reversed by therapeutic agents that regulate antioxidant pathways^113,114^. According to Kumar et al.^115^, exogenous administration of compounds that regulate proline-like chelators, reduce MDA accumulation, enhance antioxidant defense, regulate photosynthetic pigments and reduce DNA damage minimizes metal toxicity.

**Table 4:** Mitigative role of Mox against MnCl_2_-induced biochemical toxicity.

Groups	MDA (µM/g FW)	Proline (µmol/g FW)	Chlorophyll *a* (mg/g FW)	Chlorophyll *b* (mg/g FW)	SOD (U/mg FW)	CAT (OD_240 nm_ min/g FW)
C	3.75 ± 0.72^d^	11.5 ± 1.44^d^	9.46 ± 1.33^a^	4.78 ± 0.81^a^	40.6 ± 3.24^d^	0.21 ± 0.16^d^
Mox 1	3.86 ± 0.74^d^	11.1 ± 1.40^d^	9.28 ± 1.29^a^	5.00 ± 0.84^a^	38.9 ± 3.12^d^	0.19 ± 0.15^d^
Mox 2	3.68 ± 0.69^d^	11.8 ± 1.46^d^	9.55 ± 1.36^a^	4.90 ± 0.83^a^	41.7 ± 3.26^d^	0.18 ± 0.13^d^
MnCl_2_	10.9 ± 1.54^a^	30.9 ± 2.12^a^	2.85 ± 0.54^d^	1.16 ± 0.48^d^	141 ± 8.32^a^	0.44 ± 0.28^a^
MnCl_2_ + Mox 1	7.85 ± 1.36^b^	25.1 ± 1.85^b^	4.24 ± 0.76^c^	2.24 ± 0.52^c^	112 ± 6.45^b^	0.35 ± 0.24^b^
MnCl_2_ + Mox 2	5.60 ± 1.17^c^	18.2 ± 1.63^c^	6.32 ± 0.98^b^	3.50 ± 0.68^b^	80.6 ± 4.87^c^	0.29 ± 0.20^c^

C: Control, Mox 1: 250 mg/L *M. officinalis* leaf extract, Mox 2: 500 mg/L *M. officinalis* leaf extract, MnCl_2_: 1000 µM MnCl_2_, MnCl_2_ + Mox 1: 1000 µM MnCl_2_ + 250 mg/L *M. officinalis* leaf extract, MnCl_2_ + Mox 2: 1000 µM MnCl_2_ + 500 mg/L *M. officinalis* leaf extract. Data (n = 10) are displayed as mean ± standard deviation. Different letters (a–d) in the same line indicate significant differences (*p* < 0.05) between groups.

**Figure 5:**
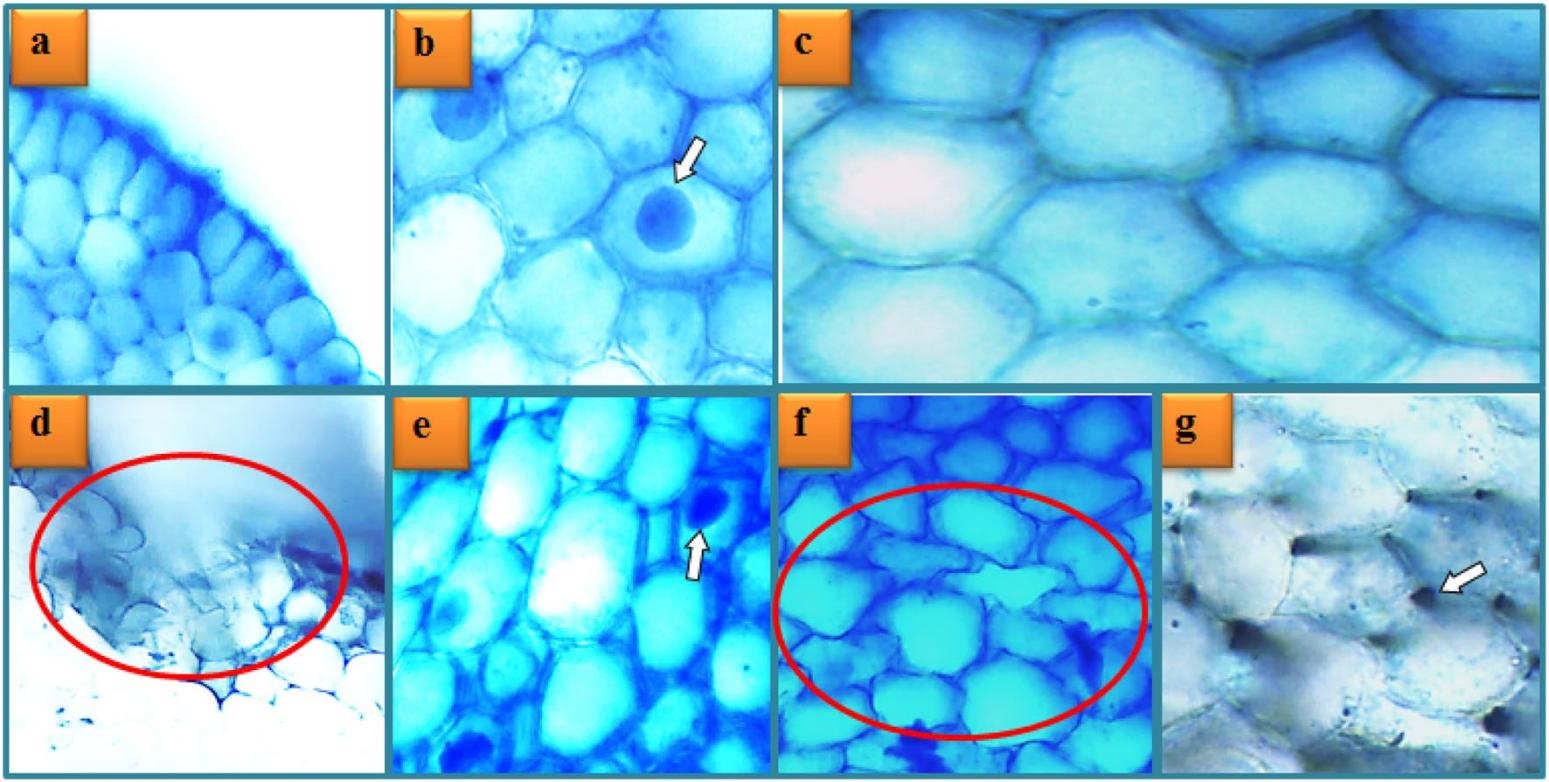
MnCl_2_-induced damage types in root meristematic cells. Epidermis normal appearance (**a**), cell nucleus normal appearance-oval (**b**), cortex normal appearance (**c**), epidermis cell damage (**d**), flattened cell nucleus (**e**), cortex cell damage (**f**), thickening of the cortex cell wall (**g**).

Table 5 demonstrates the preventive function of Mox against MnCl_2_-induced meristematic cell damage. No meristematic damage was observed in the Mox 1 and Mox 2 groups, similar to the C group. Similar to previous findings from this study, the presence of healthy, normal meristematic tissue and cell structure (Fig. 5a–c), in both the Mox 1 and Mox 2 groups demonstrates that applied doses of Mox have no toxic effects on *A. cepa* root cells. On the other hand, MnCl_2_ treatment resulted in substantial epidermis cell damage (Fig. 5d), flattened cell nucleus (Fig. 5e) and cortex cell damage (Fig. 5f), as well as moderate thickening cortex cell wall (Fig. 5g) damage. As with other heavy metals, the main target area of Mn toxicity in plants is the roots. Plants subjected to high Mn doses showed reduced root development, browning, and fissures in the roots^64^. Consistent with the MDA level, SOD and CAT activity results, epidermis cell damage and cortex cell damage are probably related to metabolic disturbance and disruption of membranes due to significant oxidative stress caused by MnCl_2_. Our study's findings corroborate those of Tümer et al.^29^, who demonstrated anatomical damages caused by Mn toxicity to *A. cepa* root cells. Additionally, Yalçın et al.^116^ identified flattened cell nucleus as a sign of genetic material damage brought on by the genotoxicity of mercury, another heavy metal. On the contrary, thickening of cortex cell wall can be a sign of compartmentalization of Mn in cell walls, which is mentioned by Li et al. ^1^ as one of the tolerance mechanisms of plants. In the MnCl_2_ + Mox 1 and MnCl_2_ + Mox 2 groups, co-application of Mox along with MnCl_2_ reduced all meristematic cell damage types. The MnCl_2_ + Mox 2 group showed the strongest relief effect; there was no thickening of the cortex cell wall and other disorders fell to "slight" levels. Reduced meristematic damage brought on by MnCl_2_-induced oxidative stress in the MnCl_2_ + Mox 1 and MnCl_2_ + Mox 2 groups can be attributed to the antioxidant abilities of the phenolic compounds of Mox.

**Table 5:** Protective role of Mox against MnCl_2_-induced meristematic cell damage.

Groups	ECD	FCD	CCD	TCCD
C	**−**	**−**	**−**	**−**
Mox 1	**−**	**−**	**−**	**−**
Mox 2	**−**	**−**	**−**	**−**
MnCl_2_	** +++ **	** +++ **	** +++ **	** ++ **
MnCl_2_ + Mox 1	** ++ **	** ++ **	** ++ **	** + **
MnCl_2_ + Mox 2	** + **	** + **	** + **	**−**

C: Control, Mox 1: 250 mg/L *M. officinalis* leaf extract, Mox 2: 500 mg/L *M. officinalis* leaf extract, MnCl_2_: 1000 µM MnCl_2_, MnCl_2_ + Mox 1: 1000 µM MnCl_2_ + 250 mg/L *M. officinalis* leaf extract, MnCl_2_ + Mox 2: 1000 µM MnCl_2_ + 500 mg/L *M. officinalis* leaf extract. ECD: epidermis cell damage, FCN: flattened cell nucleus, CCD: cortex cell damage, TCCD: thickening of the cortex cell wall (−): no damage, ( +): slight damage, (++): moderate damage, (+++): substantial damage.

## Conclusions

The objective of this research was to examine the harmful effects of Mn on the model organism *A. cepa* as well as any potential protective benefits of Mox against these adverse effects. In *A. cepa*, excessive doses of MnCl_2_ caused severe growth retardation, oxidative stress, increased cytotoxicity indicators, detrimental impacts on biochemical parameters and root meristem cell disorders. On the other hand, no toxic or genotoxic effects of the Mox doses tested on *A. cepa* were observed. Indeed, it became apparent that the detrimental effects of MnCl_2_-induced toxicity were reduced when Mox was administered together with MnCl_2_. An acknowledged eucaryotic model was used to demonstrate the harmful effects of Mn from several perspectives and to provide a starting point for future research on people. *A. cepa* was confirmed to be a trustworthy material for both toxicity and anti-toxicity assessments. The study also showed that comet and Allium assays can be efficiently used together to measure the toxicity of environmental pollutants in biological systems. Given that Mn exposure poses an increasing risk to human health, Mox has emerged as an exciting functional food option against the harmful effects of Mn with its high phenolic content and antioxidant activity. Thanks to the antioxidant and antigenotoxic properties of Mox, it has been demonstrated that it is feasible to lessen the harm brought on by heavy metal toxicity without being exposed to the side effects of drugs. Since Mox is safe when taken in appropriate dosages, its regular use should be encouraged. This work has also added to the body of knowledge that will help researchers investigate further pharmacological applications of Mox.

## Data Availability

All data generated or analyzed during this study are included in this published article.
